# Associations between childhood maltreatment and emotion processing biases in major depression: results from a dot-probe task

**DOI:** 10.1186/s12888-015-0501-2

**Published:** 2015-06-06

**Authors:** Vivien Günther, Udo Dannlowski, Anette Kersting, Thomas Suslow

**Affiliations:** LIFE-Leipzig Research Center for Civilization Diseases, University of Leipzig, Leipzig, Germany; Department of Psychosomatic Medicine and Psychotherapy, University of Leipzig, Semmelweisstr. 10, 04103 Leipzig, Germany; Department of Psychiatry, University of Marburg, Marburg, Germany; Department of Psychiatry, University of Munster, Munster, Germany

**Keywords:** Childhood maltreatment, Facial emotions, Attention, Perception, Depression

## Abstract

**Background:**

Childhood maltreatment is considered an important risk factor for the development of major depression. Research indicates an association between childhood adversity and altered emotion processing. Depression is characterized by mood-congruent cognitive biases, which play a crucial role in symptom persistence and recurrence. However, whether attentional biases in adult major depression are associated with experienced childhood neglect or abuse remains unclear.

**Methods:**

A sample of 45 patients suffering from major depression were recruited to examine correlations between maltreatment experienced during childhood and attentional biases to sad and happy facial expressions. Attention allocation was assessed using the dot-probe task and a history of childhood maltreatment was measured by means of the 25-item Childhood Trauma Questionnaire (CTQ).

**Results:**

Our results indicate an association between childhood maltreatment and sustained attention toward sad facial expressions. This relationship was not confounded by severity of symptoms, age, verbal intelligence or more recent stressful experiences.

**Conclusions:**

Our findings confirm the hypothesis that a mood-congruent bias in emotion processing observed in major depression is related to early traumatic experiences.

**Electronic supplementary material:**

The online version of this article (doi:10.1186/s12888-015-0501-2) contains supplementary material, which is available to authorized users.

## Background

Adversity and maltreatment during childhood and adolescence include forms of psychological and physical abuse, such as verbal humiliation, hostility against the child, rejection, or physical beatings, as well as sexual abuse and emotional and physical neglect [1, 2]. Prior research on relations between early adverse life events and the later development of depressive symptoms contributed to a widely accepted view that childhood maltreatment represents an important risk factor for major depressive disorder (MDD) [3, 4] (see [5, 6] for a review).

Evidence for an association between clinical depression and early emotional abuse seems to be more consistent than for relations with physical and sexual abuse [5, 7]. However, the exact mechanisms through which emotional maltreatment enhance the vulnerability to depression remain largely unknown. It has been suggested that maltreatment and negative parenting practices, such as high levels of criticism, verbal humiliation, and lack of warmth might lead to a consolidation of negative cognitive thinking styles [8]. A growing body of research has provided empirical support for this assumption [9–12], see [13] for a review. Negative cognitive styles, such as dysfunctional attitudes and negative attribution styles are considered important vulnerability factors for depression [14, 15]. Furthermore, according to cognitive theories, negatively biased processing of emotional information enhances susceptibility for developing depression and accounts for symptom persistence and recurrence [14]. In line with these theories, a wealth of research found that depressed individuals differ from healthy controls in their processing of emotional material [16, 17]. Depressed patients show negative biases in perception and interpretation of environmental information, attention, and memory [18–21]. Gotlib et al. [22] reported difficulties in disengaging attention from sad faces in depressed patients compared to healthy controls using a modified version of the dot-probe task. This reaction time task was developed to draw conclusions about visual attention allocation [23]. The finding of sustained attention toward negative stimuli in depressed patients was replicated by several authors [24–26]. Studies using neuroimaging techniques revealed a hyper-responsiveness of the amygdala to negative stimuli in patients suffering from MDD [27–31]. The amygdala plays a key role in the processing of emotional stimuli and in enhancing levels of attention toward these stimuli [32, 33]. Its involvement in the production of negative affective states has been well documented [34]. Thus, amygdala hyperactivity has been considered as a possible neural underpinning of negative cognitive biases observed in MDD [35, 36].

A history of childhood maltreatment seems to moderate higher amygdala reactivity in MDD [37]. Physical abuse was positively correlated with amygdala responsiveness toward sad faces within depressed patients. Furthermore, no group differences were found for amygdala reactivity between healthy control subjects and depressed patients without a history of maltreatment during childhood. In line with this finding, van Harmelen et al. [38] reported enhanced amygdala reactivity to negative stimuli in emotionally maltreated adults independent of their psychiatric status. Using subliminally presented sad faces, Dannlowski et al. [39] found associations between childhood maltreatment and amygdalar hyper-activation in a large sample of participants without any psychiatric conditions. It remains to be investigated, whether neural alterations in maltreated individuals underlie behavioral biases in emotion perception.

Only few psychological studies investigated effects of child maltreatment on processing of emotional cues. Healthy children and adults exposed to maltreatment exhibited a greater sensitivity in detecting threatening cues from emotionally ambiguous faces [40, 41], but needed more facial information to correctly detect expressions of sadness [41]. These results indicate a facilitated processing of threatening stimuli and impairments in detection of sad faces in abused individuals. There is evidence for associations between early adverse experiences and attentional biases for threatening faces [40, 42], however see [43] for contradictory results. In sum, previous research suggested atypical patterns in processing of threatening information among maltreated individuals.

However, depression is characterized by attentional biases to emotionally congruent stimuli such as sad faces or depression-related words [44, 45]. Furthermore, there is evidence for a lack of attentional bias toward positive stimuli [45]. Few studies illuminated the relationship between childhood maltreatment and biased attention to sad stimuli, whereas it seems important to use stimuli relevant for depression when investigating vulnerability to this disorder. Gibb et al. [40] compared students with and without a history of any form of psychological or physical maltreatment and failed to find group differences in attentional biases toward sad faces. However, expanding these results, Romens and Pollak [46] reported difficulties in disengaging attention from sad faces among healthy abused children, but only during the recovery phase after a sad mood induction or among those maltreated individuals engaged in habitual rumination.

In general, the contradictory results for attentional biases to facial emotions as a function of childhood maltreatment might be explained by strong variations in sample characteristics such as age and psychopathological status and differences in terms of the experimental tasks. Several of the aforementioned studies used long stimulus presentation times whereas other studies administered rather short ones. In depression, there is growing evidence for attentional biases for long stimulus presentation durations [22, 24, 47, 48]. These findings indicate that depressed individuals show primarily impaired disengagement of attention from negative stimuli. Thus, longer presentation durations for depression-related stimuli might be necessary to detect abnormal patterns in attentional control among maltreated individuals.

In our study, we examined attentional biases in response to sad and happy facial expressions as a function of childhood maltreatment in individuals suffering from MDD. To our knowledge, no previous study explored relations between child maltreatment and biased attention in adult depression. We administered the dot-probe task and a questionnaire to assess early childhood experiences to an inpatient group. We expected patients reporting childhood maltreatment to exhibit a stronger attentional bias toward sad faces independent of their symptom severity. In line with findings indicating an attentional avoidance of positive stimuli in depressed patients [45], we expected an inverse relationship among childhood maltreatment and bias to happy faces.

## Methods

### Participants and psychometric measures

Our participants were 45 inpatients (30 female, 15 male) recruited from a treatment program of the Department for Psychosomatic Medicine and Psychotherapy of the University of Leipzig. Age of participants ranged between 19 and 55 years. Demographic, questionnaire and clinical sample characteristics are presented in Table 1.

**Table 1:** Demographic, questionnaire and clinical sample characteristics (means and SD (in brackets))

Variable	
Age	34.04 (9.48)
Level of education^a^	2.76 (0.98)
*N* no degree	0 (0 %)
*N* 9th grade	3 (6.7 %)
*N* 10th grade	18 (40.0 %)
*N* 12th grade	12 (26.7 %)
*N* university degree	11 (24.4 %)
*N* PhD	1 (2.2 %)
Verbal intelligence	111.40 (13.42)
Duration of current episode of illness (in months since symptom onset)	8.00 (13.13)
Number of episodes	2.95 (2.20)
Age at onset of first episode	26.53 (9.99)
Lifetime hospitalization (weeks)	5.62 (8.52)
HAMD	14.44 (3.92)
BDI-II	30.62 (9.52)
BAI	26.80 (9.86)
CTQ total score	54.02 (19.23)
CTQ emotional abuse	12.02 (5.95)
CTQ physical abuse	8.36 (5.56)
CTQ sexual abuse	7.31 (5.51)
CTQ emotional neglect	16.89 (5.31)
CTQ physical neglect	9.44 (3.53)
PSS	39.56 (4.12)
LTE-Q	2.80 (1.84)

^a^Coding of level of education: 0 = no degree, 1 = 9^th^ grade, 2 = 10^th^ grade, 3 = 12^th^ grade, 4 = university degree, 5 = PhD; HAMD, Hamilton Depression Scale; BDI-II, Beck Depression Inventory; BAI, Beck Anxiety Inventory; CTQ, Childhood Trauma Questionnaire; PSS, Perceived Stress Scale; LTE-Q, List of Threatening Experiences Questionnaire

The Structured Clinical Interview for DSM-IV Axis I disorders (SCID-I) [49] was administered to determine diagnosis of MDD. Twenty-nine of the depressed patients met criteria for comorbid anxiety (panic disorder, agoraphobia, social phobia, specific phobia, obsessive-compulsive disorder, posttraumatic stress disorder and anxiety disorder not otherwise specified), somatoform disorders (pain disorder and undifferentiated somatoform disorder) or eating disorders (bulimia nervosa and binge eating disorder). In the context of the SCID-I interview, 10 patients reported to have experienced trauma during adulthood, such as car accidents, threats or physical violence, and sexual assaults. One of these patients was diagnosed with posttraumatic stress disorder. Exclusion criteria were any history of bipolar or psychotic disorders and substance abuse or addiction within the previous six months. Thirty-two patients were taking antidepressant medication, 2 were additionally treated with benzodiazepines. Severity of depressive symptoms was assessed with the revised version of the Beck Depression Inventory (BDI-II, German version [50]) and the Hamilton Depression Scale (HAMD, German version [51]). Total scores of BDI and HAMD range from 0 to 63 and from 0 to 52, respectively, with higher scores indicating more severe symptoms. Level of current anxiety was evaluated by the Beck Anxiety Inventory (BAI, German version [52]). A total score between 0 and 63 can be achieved, with higher scores indicating more pronounced anxiety symptoms. Trauma exposure during childhood was measured with the German version of the Childhood Trauma Questionnaire (CTQ), a retrospective self-report measure consisting of 25 items [53]. The CTQ has five subscales, each comprised of five items, assessing emotional, physical and sexual abuse and emotional and physical neglect. All items are rated on a five-point Likert scale (1 = “applies not at all” to 5 = “applies entirely”). Scores of the total scale range from 25 to 125, and scores of subscales from 5 to 25, with higher scores indicating more severe abuse or neglect. The CTQ is a well-validated instrument showing high psychometric properties in healthy subjects and clinical samples [53–55]. Internal consistency was good in the present study (all Chronbach’s α’s > .88) with exception for the physical neglect subscale (Chronbach’s α = .64). Patients also completed the 10-item version of the Perceived Stress Scale (PSS) [56] to measure subjectively experienced stress during the past month, indicated by the degree to which individuals appraised situations in life as uncontrollable, unpredictable and overburdening [57]. Total scores of the PSS can range from 10 to 50. As an objective measure of recent stress, the List of Threatening Experiences Questionnaire (LTE-Q) [58] was applied. The LTE-Q total scores have a range from 0 to 12. The LTE-Q assesses the occurrence of 12 life events during the last 12 months, that have noticeable long-term threat, particularly on mental health [58, 59]. The LTE-Q encompasses life events such as severe illness, loss of close family members, financial problems or unemployment.

Patients’ verbal intelligence was assessed by means of the Mehrfachwahl-Wortschatz-Intelligenztest (MWT-B), a multiple choice test using artificial and existent vocabulary of the German language [60].

The study was approved by the local ethics committee of the University of Leipzig. After a detailed explanation of the study, written informed consent was obtained from all participants and they received financial compensation after completion of all tasks.

### Procedure

Following the SCID-I and HAMD interview during the first session, patients who met inclusion criteria were scheduled for the second experimental session within one week. (With exception of one patient, who completed the second session after two weeks, due to the occurrence of minor health issues). During the second session, participants completed the self-report questionnaires and afterwards the computer-based dot-probe task.

### Measurement of attentional biases

The dot-probe task was administered to assess patients’ attentional biases to sad and happy facial expressions. Stimuli for the dot-probe task were colored photographs of 40 actors (20 male, 20 female) depicting happy, sad and neutral facial expressions. Pictures were obtained from the Karolinska Directed Emotional Face database (KDEF) [61]. At the beginning of the experiment each neutral face (n = 40) was randomly paired with either the happy or the sad facial expression of the same actor. Thus, stimuli consisted of pairs of photographs of an actor, one depicting a neutral facial expression and the other an emotional facial expression. The neutral face was assigned to the left or the right side of the screen with equal frequency. Each pairing was presented twice and in random order, resulting in a total of 80 experimental trials. Presented on a 19 inch monitor, the size of each face picture was approximately 10.4 cm × 11.8 cm. The centers of both pictures were approximately 18.5 cm apart.

Each trial of the dot-probe task began with a fixation cross presented for 500 ms in the center of the screen. It was followed by the paired photographs. After 1000 ms both pictures disappeared and were replaced by an asterisk appearing either on the left or right position of the face photographs. For each emotion (sad and happy) the asterisk substituted the emotional and neutral face with equal frequency. Participants were instructed to indicate the position of the asterisk (left vs. right) as quickly as possible by a button press on a keyboard. Therefore, the left and right index fingers were used. The asterisk remained on the screen until a response was given. The inter-trial interval was 1000 ms.

Trials with response errors (1.6 %) and reaction times below 100 ms and greater than 1000 ms (0.6 %) were excluded from analyses. Using the equation of Mogg et al. [62], attentional biases toward emotional faces compared to neutral faces were computed separately for each emotion (happy and sad). Mean latencies for trials in which the probe appeared in the location of the emotional face (congruent condition) were subtracted from trials in which the probe replaced the neutral face (incongruent condition). The dot-probe task is based on the assumption that responses are faster when the probe appears at the previously attended location. Higher bias scores indicated preferential attention toward emotional faces compared to neutral faces whereas negative scores indicate attentional avoidance of emotional faces. Mean reaction times for the different experimental conditions are presented in Table 2.

**Table 2:** Reaction times for each emotion type for congruent and incongruent trials in the dot-probe task

Emotion type	Condition	*M* (*SD*)
Happy-neutral	Congruent	395.47 (69.05)
	Incongruent	394.35 (74.00)
Sad-neutral	Congruent	398.60 (79.28)
	Incongruent	395.76 (73.19)

Pearson product–moment correlations were conducted to examine associations between the CTQ scales and attentional bias scores separately for happy and sad facial expressions. To assess possible differences in the strength of correlations between biases and CTQ subscales, Steiger’s *Z* was computed using formulas provided by Lee and Preacher [63].

A set of subsequent two-stage hierarchical regression analyses was calculated with attentional bias as dependent variable. This method was chosen to control for potential modulatory effects of illness severity, current anxiety level, verbal intelligence, age and recent stressful experiences on the relationship between childhood maltreatment and attentional biases. Therefore, scores of the BDI, HAMD, BAI, MWT-B, PSS and LTE-Q, as well as age were entered as predictors in the first step of the regression model to regress out their possible influence on attentional biases. In a second step, the scales of the CTQ were entered as predictors of interest. Hierarchical regression analyses were calculated only for those CTQ scales showing significant associations with attentional bias scores in the previous correlation analyses. For all scales of the CTQ and attentional bias scores there were no differences between men and women, no differences in medicated compared to unmedicated patients and no differences between depressed patients with and without comorbid diagnoses (all *p*s > .13). Thus, we did not include gender, comorbidity and medication status as predictors in the hierarchical regression model. To account for multiple testing a conservative significance level of *p* ≤ .01 was used for correlation and regression analyses and computations of Steiger’s *Z*.

## Results

**Fig. 1:**
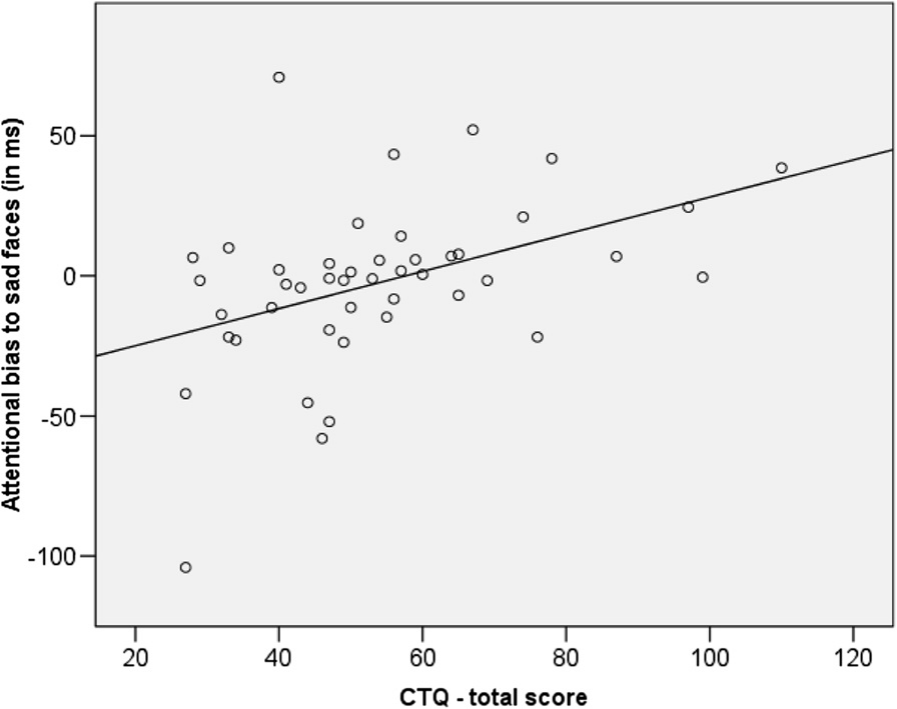
Relationship between total CTQ score with attentional bias toward sad facial expression. (*r* = .43, *p* < .01, two-tailed)

Correlation analyses yielded a positive association between the total CTQ score and attentional bias toward sad facial expression (see Fig. 1). Individuals scoring higher on the childhood trauma scale exhibited stronger attentional bias to sad faces. Table 3 presents intercorrelations for all variables of interest. Of all five subscales, only emotional abuse and physical neglect were significantly correlated with attentional bias scores for sad faces. Additionally, analyses revealed a marginally significant correlation between attentional bias scores for sad faces and emotional neglect. Emotional abuse yielded the strongest correlation, followed by physical neglect and emotional neglect. However, according to Steiger’s *Z* there were no significant differences in the strengths of correlations with the different CTQ subscales when accounting for multiple testing (all *p*s > .04).

**Table 3:** Pearson product–moment correlations between childhood trauma scales and attentional biases

	Bias		CTQ				
	Sad	Happy	Total	EA	PA	SA	EN
Bias sad	-						
Bias happy	-.38**	-					
CTQ-total	.43**	-.28	-				
CTQ-emotional abuse	.47**	-.33*	.86**	-			
CTQ-physical abuse	.22	.02	.82**	.62**	-		
CTQ-sexual abuse	.18	-.19	.52**	.29	.37*	-	
CTQ-emotional neglect	.33*	-.31*	.74**	.61**	.48**	.02	-
CTQ-physical neglect	.43**	-.23	.79**	.64**	.54**	.19	.71**

*p < .05 two-tailed; **p < .01 two-tailed; CTQ, Childhood Trauma Questionnaire

Furthermore, results indicated marginally significant negative relationships between emotional maltreatment (abuse and neglect) and attentional bias for happy faces. Here, higher scores in both CTQ subscales predicted lower scores in attentional bias to happy faces. Computing Steiger’s *Z*, only the strength of correlation with bias to happy faces and emotional abuse differed significantly from the correlation with physical abuse (*p* < .01).

Notably, severity of depressive symptoms was neither correlated with attentional bias to sad (for BDI, *r* = .14, *p* = .37 and for HAMD, *r* = .06, *p* = .67) nor with attentional bias to happy faces (for BDI, *r* = −.04, *p* = .80 and for HAMD, *r* = −.25, *p* = .09).

In the first step of hierarchical regression analyses, variance in attentional biases for sad faces was not significantly explained by any predictor (all *p*s > .16, see Additional file 1), *R*^*2*^ = .08; *F*(7,44) = 0.46, *p* = .86). Thus, severity of depression, anxiety, verbal intelligence, age, and recent stressful life events did not predict attentional bias to sad faces. However, entering the total score of the CTQ in the second step did significantly increase the predictive value of the model (*ΔR*^*2*^ = .23, *p* < .01; *F*(8,44) = 2.05, *p* = .07; see Table 4). The same pattern of results was yielded for the CTQ subscales of emotional abuse (*ΔR*^*2*^ = .25, *p* < .01; *F*(8,44) = 2.19, *p* = .05) and physical neglect (*ΔR*^*2*^ = .23, *p* < .01; *F*(8,44) = 1.98, *p* = .08). Both factors of the CTQ enhanced the explained variance in attentional bias to sad faces significantly. Hence, the predictive value of childhood maltreatment for attentional biases toward sad faces remained significant after controlling for the potential influence of symptomatology, verbal intelligence, age, and recent stressful life events. Correlations between attentional bias to sad faces and emotional neglect and between attentional bias to happy faces and emotional maltreatment (abuse and neglect) did not survive correction for multiple testing. Thus, we excluded these CTQ scales from regression analyses.

**Table 4:** Hierarchical regression analyses with attentional bias to sad facial expressions as dependent variable

	Attentional bias sad			
	*β*	*R* ^*2*^	*ΔR* ^*2*^	*Partial η* ^*2*^
*Step 1*		.08	.08	
-				
*Step 2*		.31	.23**	
CTQ-total	.54**			.25
*Step 2*		.33	.25**	
CTQ-emotional abuse	.56**			.27
*Step 2*		.31	.23**	
CTQ-physical neglect	.55**			.25

Note: Only regressors with *p* < .1 are reported, but see Additional file 1 for a detailed list of all regressors. *p < .05; **p < .01; CTQ, Childhood Trauma Questionnaire

## Discussion

The aim of our study was to investigate attentional biases to sad and happy facial expressions as a function of childhood maltreatment in individuals suffering from MDD. To our knowledge, this was the first study examining the relations between early adverse experiences and attention to sad and happy faces in adult depression. Hence, we extended previous research on associations between childhood maltreatment and emotion processing biases in healthy individuals [40–42] and individuals with mild self-rated symptoms of posttraumatic stress disorder [43]. Results confirmed our hypotheses regarding a relation between childhood maltreatment and attentional bias toward sad facial expressions. Individuals reporting more severe maltreatment during childhood maintained their attention to sad faces and thus, showed a stronger mood-congruent bias. Analyzing the five factors of childhood maltreatment separately, associations were significant only for emotional abuse and physical neglect, marginally significant for emotional neglect, but not for physical and sexual abuse. Mean scores of emotional neglect and sexual and physical abuse scales were comparable to other clinical samples [37, 64] and variance in all measures was similar to the other CTQ subscales within our sample (see Table 1). Hence, the lack of significant correlations for emotional neglect and sexual and physical abuse might not be explained by restricted variability in values or atypical prevalence of these forms of maltreatment in the present sample.

According to hierarchical regression analyses childhood maltreatment is related to attention bias toward sad faces after controlling for possible mediating effects of current depression and anxiety symptoms, verbal intelligence, age, subjectively experienced stress during the past month or the occurrence of stressful life events within the last year. Nevertheless, it is important to note that the majority of patients received antidepressant medication and we could not control for possible influences of different dosages or types of medication on attentional biases. Gibb et al. [40] found no group differences in attentional biases for sad faces between undergraduates with and without experienced abuse. However, in line with our results, abused children exhibited sustained attention to sad faces during the recovery from a sad mood induction [46]. Mood deterioration was previously shown to be sufficient for the emergence of negative cognitive biases in individuals prone to depression [65-67]. According to Beevers’ [68] vulnerability model for depression, individuals at risk might be able to inhibit biases in emotional processing, but only when cognitive resources for reflective processing are available. Moreover, cognitive theories of depression suggest that dysfunctional cognitive schemas, manifested through adverse early life experiences, remain inactive in the non-depressed state, but can be triggered by life events [14]. Hence, it has been proposed that interindividual differences in cognitive biases might only emerge when relevant schemas are activated or primed, for example by sad mood or stressful experiences [66, 69]. Thus, depression-relevant behavioral biases in maltreated individuals might be detectable only under certain circumstances, such as depressed mood, after stressful life events, or under high cognitive load. Given that we did not include a healthy control group in our study to test for this hypothesis, our assumptions are only speculative and require further investigation. We demonstrated a relation among childhood maltreatment and altered attention to sad faces in clinical depression. The lack of similar findings in previous studies with non-depressed subjects might also be due to methodological differences or insufficient sample sizes to detect small effects. Further evidence for the occurrence of biases under restricted conditions was provided by Wells and colleagues [70]. Examining interpretation biases for ambiguous sentences, associations with childhood physical maltreatment were only significant under a cognitively demanding condition. Automatic negative cognitive biases in maltreated individuals were not observable when cognitive resources allowed for an effortful correction.

Mood-congruent biases in processing of depression-related stimuli have been repeatedly observed in patients suffering from MDD compared to healthy controls. Negative biases in emotion processing have been discussed as a cognitive vulnerability factor for the development, maintenance, and recurrence of depressive symptoms [14]. Our results provide further support for the hypothesis that childhood maltreatment may be a factor contributing to the consolidation of mood-congruent biases in emotion processing. However, whether this negative bias can be considered as a risk factor, manifested before the development of a depressive episode, or is a consequence of suffering from depression, remains unclear. Longitudinal research found increases in depressive inferential styles and rumination in children experiencing emotional maltreatment [9, 11, 71]. Future longitudinal studies have to examine influences of childhood maltreatment on the subsequent development of mood-congruent attentional biases.

Neuroimaging studies have provided evidence for a negatively biased emotion processing in limbic brain regions, such as the amygdala, as a function of early experienced maltreatment [37–39, 72]. Only few studies have investigated brain activation patterns that are associated with cognitive biases in depression (see [73] for a review). High reactivity of the amygdala to negative stimuli in depressed patients was found to be associated with negative evaluative biases [36] and negative memory biases [74]. Considering its role in recruiting attentional resources and directing attention toward emotional stimuli [32, 33], hyper-responsiveness of the amygdala might be a neural mechanism exerting influence on negatively biased attention. Thus, enhanced amygdala activity to negative stimuli observed in maltreated individuals might be related to attentional biases found in our study. Further research is needed to examine this relationship among depressed individuals with respect to the possible moderating role of childhood maltreatment.

Our data also suggest a non-significant trend toward an inverse association between emotional maltreatment and attentional biases to happy faces. This negative relationship denotes an attentional avoidance of positive facial emotions in individuals with a more severe emotional maltreatment history. Two previous studies already documented non-significant trends to avoiding happy faces among abused students [40], and among maltreated children reporting high levels of rumination [46]. Hence, in future studies larger sample sizes are required to reliably detect associations between childhood maltreatment and avoidance of happy facial expressions. Several research groups reported favored processing of positive stimuli, a so called “protective” or positive perceptual bias, in healthy control subjects compared to depressed patients [25, 75–77]. Thus, not only negative biases but also the absence of preferential processing for positive stimuli seems to be a feature of depressive perception [45]. In our study, specifically those patients reporting emotional maltreatment during childhood tended to lack a processing advantage for positive stimuli. However, it must be noted that these correlations did not survive correction for multiple testing.

Regarding mood-congruent attentional biases to emotional faces, our study identified a stronger link to emotional maltreatment and physical neglect, rather than to physical or sexual abuse. This finding is in line with the assumption of Rose and Abramson [8] that particularly emotional abuse might lead to the development of depressive cognitive styles. Moreover, stronger empirical support exists for a relation between emotional abuse in childhood and the later development of depressive symptoms than for other forms of early adverse life events [5]. Only emotional maltreatment was found to be significantly associated with automatic depressive self-associations [78] and negative inferential styles [10]. In contrast, different research groups reported relations between physical maltreatment and a biased processing of threatening information [40–42]. We did not include threat-related stimuli, such as angry or anxious faces, in our dot-probe task. This might be a possible reason why no relations between attentional biases and physical abuse were observed in our study. Another explanation might be our relatively small sample size and the lack of power to reveal rather small correlations. Furthermore, it must be acknowledged that analyses did not reveal significant differences in the strength of correlations between CTQ subscales and attentional bias to sad faces.

Some further limitations of our study must be noted. Our depressed patients suffered also from comorbid anxiety, somatoform, and eating disorders. We did not find statistical evidence for confounding effects of comorbidity, but these analyses might be underpowered due to our small sample size. Anxiety disorders are frequently coexisting with depression [79]. Our sample might be rather representative for inpatients suffering from clinical depression. The majority of our patients were treated with antidepressant medication. We documented dosage and treatment duration, but we were not able to code medication levels in terms of antidepressant potency according to Sackeim [80]. Several recently released antidepressants are not specified in the rating assignments. Thus, we could not statistically control for possible influences of medication. Furthermore, our cross-sectional design and the assessment of childhood maltreatment by means of a retrospective self-report measure do not allow drawing conclusions about the causal relationship between early adverse life events and biased emotion processing. Nevertheless, depression theories have proposed that childhood maltreatment may contribute to the development of negative biases [8, 14]. In our study, patients with a current negative attentional focus might have recalled more negative memories from childhood and thus, reported more severe maltreatment experiences. In future studies, a more objective rating of childhood maltreatment, using information from external sources, could improve the strength of conclusions. However, numerous studies confirmed the good psychometric properties of the CTQ and the measure has been widely used in childhood trauma research [2, 53, 55, 81, 82]). We measured recent stressful experiences, but we did not assess past exposure to traumatic events. Future studies should control for the possible influence of more recent traumatic experiences. According to our HAMD scores, severity of depressive symptoms was relatively low in the present sample. Usually, our patients are subject to a waiting period until admission to our clinic. Serious suicidal intentions or suicide attempts are general contraindications for admission. The treatment program of the department is especially suited for patients suffering from moderately severe depressive symptoms. Thus, no severe cases of acute depression were included in our sample.

## Conclusion

In sum, our results provide support for the assumption that mood-congruent biases in emotion processing are associated with traumatic childhood experiences in depression. It has been argued that negative cognitive biases play an important role in the maintenance and recurrence of depressive symptoms and might be relevant for treatment outcome [14]. There is evidence for a worse course of illness and higher recurrence of symptoms among individuals exposed to childhood maltreatment [83]. It can be assumed that negative emotion processing biases might contribute to poor treatment response of depressed patients with a history of childhood maltreatment as mediating factors.

## Additional file

Additional file 1:
**Detailed listing of regressors for hierarchical regression analyses with attentional bias to sad facial expressions as dependent variable.**

## Abbreviations

BAI: Beck anxiety inventory
BDI-II: Beck depression inventory
CTQ: Childhood trauma questionnaire
EA: Emotional abuse
EN: Emotional neglect
HAMD: Hamilton depression scale
LTE-Q: List of threatening experiences questionnaire
M: Mean
MDD: Major depressive disorder
PA: Physical abuse
PN: Physical neglect
PSS: Perceived stress scale
SA: Sexual abuse
SD: Standard deviation
