# Supplementary material for: Associations between childhood maltreatment and emotion processing biases in major depression: results from a dot-probe task
Source: BMC Psychiatry. 2015 Jun 6;15:123. doi: 10.1186/s12888-015-0501-2 (PMC4458030; doi:10.1186/s12888-015-0501-2)
Supplement: Additional file 1: — Detailed listing of regressors for hierarchical regression analyses with attentional bias to sad facial expressions as dependent variable. [file 12888_2015_501_MOESM1_ESM.docx]

Additional File 1

Detailed listing of regressors for hierarchical regression analyses with attentional bias to sad facial expressions as dependent variable

|  | attentional bias sad | | |  |
| --- | --- | --- | --- | --- |
|  | *β* | *R^2^* | *ΔR^2^* | *partial η^2^* |
| *step 1* |  | .08 | .08 |  |
| HAMD | .02 |  |  | .00 |
| BDI-II | .29 |  |  | .05 |
| BAI | -.08 |  |  | .01 |
| Verbal Intelligence | .08 |  |  | .01 |
| PSS | -.22 |  |  | .03 |
| LTE-Q | -.03 |  |  |  |
| Age | .05 |  |  | .00 |
| *step 2* |  | .31 | .23** |  |
| HAMD | -.05 |  |  | .00 |
| BDI-II | .19 |  |  | .03 |
| BAI | -.21 |  |  | .04 |
| Verbal Intelligence | .16 |  |  | .03 |
| PSS | -.21 |  |  | .04 |
| LTE-Q | -.09 |  |  | .01 |
| Age | .05 |  |  | .00 |
| CTQ-Total | .54** |  |  | .25 |
| *step 2* |  | .33 | .25** |  |
| HAMD | -.15 |  |  | .03 |
| BDI-II | .17 |  |  | .03 |
| BAI | -.17 |  |  | .03 |
| Verbal Intelligence | .12 |  |  | .02 |
| PSS | -.24 |  |  | .05 |
| LTE-Q | -.02 |  |  | .00 |
| Age | .03 |  |  | .00 |
| CTQ-Emotional Abuse | .56** |  |  | .27 |
| *step 2* |  | .31 | .23** |  |
| HAMD | .00 |  |  | .00 |
| BDI-II | .12 |  |  | .01 |
| BAI | -.13 |  |  | .02 |
| Verbal Intelligence | .27 |  |  | .06 |
| PSS | -.13 |  |  | .01 |
| LTE-Q | -.08 |  |  | .01 |
| Age | -.11 |  |  | .01 |
| CTQ-Physical Neglect | .55** |  |  | .25 |

HAMD, Hamilton Depression Scale; BDI-II, Beck Depression Inventory; BAI, Beck Anxiety Inventory; CTQ, Childhood Trauma Questionnaire; PSS, Perceived Stress Scale; LTE-Q, List of Threatening Experiences Questionnaire
